# Water‐Activated Phospholipid Phase‐Separation Gel as an Immunomodulatory Anti‐Adhesion Barrier for Postoperative Adhesion Prevention

**DOI:** 10.1002/adhm.71710

**Published:** 2026-09-09

**Authors:** Jong‐Ju Lee, Yulseung Sung, Yeji Kim, Heesang Yang, Suyeon Pyo, Byeol Yun, Sung‐Joo Hwang

**Affiliations:** ^1^ Yonsei Institute of Pharmaceutical Sciences College of Pharmacy Yonsei University Incheon Republic of Korea; ^2^ Boston Children's Hospital and Harvard Medical School Boston Massachusetts USA; ^3^ Department of Integrative Biotechnology Yonsei University Incheon Republic of Korea

**Keywords:** immunomodulation, phospholipid phase‐separation gel, postoperative adhesion prevention, surgical barrier, water‐activated gelation

## Abstract

Postoperative adhesions remain a major complication after abdominal surgery, and current polymeric barriers often show limited tissue retention and biocompatibility. Here, we establish a water‐activated phospholipid barrier strategy for postoperative adhesion prevention by converting a depot‐forming phospholipid phase‐separation gel (PPSG) into a gram‐scale surgical barrier. In this study, solvent identity acts as a functional design determinant rather than a passive solubilizer, governing hydration‐triggered gel reinforcement, intraperitoneal residence, and tolerability. Replacing ethanol with propylene glycol strengthened the gel matrix, improved barrier activation, and enabled gram‐scale administration without hepatic toxicity associated with ethanol‐based PPSG. Mechanistically, the structured phospholipid barrier retained gel‐state immunomodulatory activity, attenuating macrophage‐driven inflammatory responses and suppressing NF‐κB‐associated signaling. In a rat peritoneal‐cecum abrasion model, propylene glycol‐based PPSG completely prevented macroscopic adhesions. Together, these findings establish a water‐activated phospholipid barrier platform for converting depot‐forming phospholipid systems into active surgical barriers that combine durable physical separation with local immunomodulation.

## Graphical abstract

A water‐activated phospholipid phase‐separation gel is repurposed as a drug‐free surgical barrier for postoperative adhesion prevention. Propylene glycol promotes rapid lamellar gel reinforcement, prolonged local retention, and tissue coverage, while phosphatidylcholine‐mediated immunomodulation suppresses inflammatory and fibrotic responses. This dual‐action barrier prevents postoperative adhesions without requiring an additional therapeutic agent.

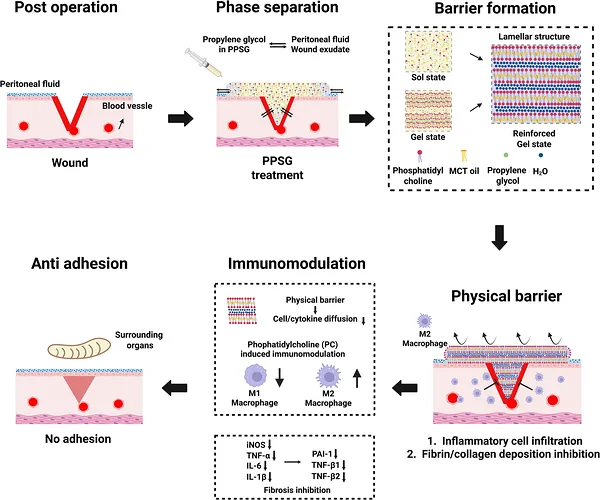

## 1 Introduction

Postoperative adhesions arise when injured intra‐abdominal tissues abnormally adhere during wound healing, often leading to bowel obstruction, chronic pain, and infertility [1, 2, 3]. The incidence of peritoneal adhesions can reach up to 89% in individuals with a history of abdominopelvic surgery [4, 5, 6]. In addition, in patients who develop adhesions, re‐operations are associated with prolonged operative time and worse surgical outcomes, leading to higher medical costs [4].

To reduce adhesion formation, several barrier materials have been translated clinically, including hyaluronic acid‐based or carboxymethyl cellulose‐based hydrogels/films and poloxamer‐based thermosensitive hydrogels [7, 8, 9]. However, the clinical performance of these materials can be limited by short residence time at the injury site, insufficient mechanical stability, and incomplete coverage of irregular tissue surfaces. Moreover, physical barrier formation alone may not fully address the inflammatory and fibrotic processes involved in adhesion formation. To improve barrier performance, recent studies have introduced reinforced polymer networks, crosslinking strategies, or anti‐inflammatory and anti‐fibrotic agents [10, 11, 12, 13]. These approaches can improve material performance but may also increase formulation complexity and require further evaluation of degradation behavior, safety, and translational feasibility.

Over the past few decades, phospholipid phase‐separation gel (PPSG) has been studied as a drug‐delivery platform that can serve as an alternative to polymer‐based in situ‐forming delivery systems. When administered as a low‐viscosity sol, PPSG is activated by physiological fluids and undergoes a hydration‐triggered sol to gel transition to form a reinforced matrix. This phase transition enables sustained local delivery of cargos ranging from small molecules to peptides and proteins [14, 15, 16, 17] (Figure 1A). Conventional PPSG consists of phospholipids, pharmaceutical oil, and a small amount of ethanol, offering low‐cost components, biodegradability, and scalability using FDA‐approved excipients [18].

**FIGURE 1:**
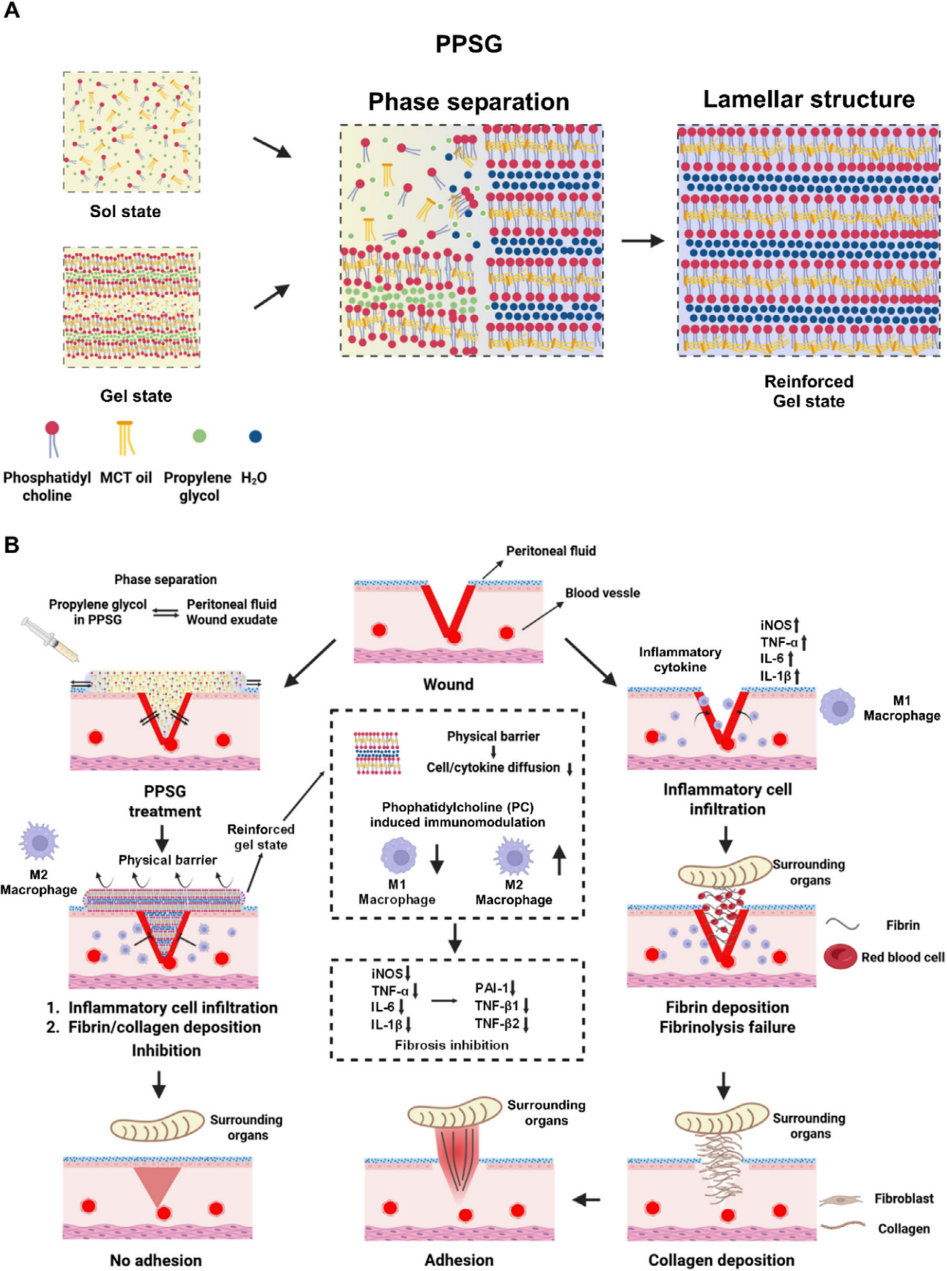
Mechanism of PPSG for postoperative adhesion prevention. (A) Schematic illustration of the water‐activated sol‐gel transition in propylene glycol‐based PPSG. (B) Dual‐function mechanism of PPSG combining physical barrier formation and anti‐inflammatory effects.

Phase separation has been widely used to design functional gel materials with tunable mechanical and interfacial properties. Most reported phase‐separated hydrogels and ionogels rely on polymer networks as the structural framework, in which polymer‐rich and polymer‐poor domains or polymer–solvent demixing are used to regulate mechanical reinforcement, lubrication, interfacial wetting, or stimulus‐responsive functions [19, 20, 21, 22]. In contrast, PPSG is formed from non‐polymeric phospholipid components, pharmaceutical oil, and solvents, and its phase transition is governed by solvent exchange and phospholipid molecular reorganization upon contact with physiological fluid. Rather than relying on phase separation within a preformed polymer network, PPSG forms a lamellar gel matrix through the rearrangement of phospholipid assemblies. This distinction enables in situ barrier formation without covalent crosslinking or additional polymer network construction.

Building on this solvent‐exchange‐driven phospholipid assembly, we sought to re‐engineer PPSG for gram‐scale use at surgical sites exposed to physiological fluid, where rapid in situ activation, stable surface coverage, barrier mechanics, and excipient tolerability are important design requirements. Since the barrier matrix is phosphatidylcholine‐rich, we considered prior reports showing that phosphatidylcholine attenuates lipopolysaccharide (LPS) induced inflammatory responses in multiple systems [23, 24, 25], providing a rationale for evaluating local immunomodulatory effects in our system. Consistent with this rationale, anti‐adhesion materials that couple physical separation with anti‐inflammatory effects has shown improved outcomes in preclinical studies [26, 27]. Thus, PPSG may serve as a dual‐functional physical barrier that both physically separates injured tissues and attenuates inflammation, without the need for an additional active pharmaceutical ingredient (API) (Figure 1B). These considerations raise two key questions: whether a structured phospholipid gel can retain immunomodulatory bioactivity in vivo, and whether conventional ethanol‐containing PPSG can be safely deployed at the gram scale required for intra‐abdominal barrier applications.

A central challenge in this conversion is that the solvent in PPSG is not merely a diluent. Rather, it governs hydration‐triggered activation kinetics, phase‐transition behavior, barrier mechanics, residence, and safety in vivo. Conventional ethanol‐containing PPSGs were developed for low‐volume depot applications, whereas intra‐abdominal barrier use requires gram‐scale administration and thereby raise distinct tolerability concerns, particularly because local ethanol exposure can impair wound healing and high ethanol exposure has been associated with hepatotoxicity [28, 29, 30]. Here, using solvent‐matched PPSGs, we define a solvent‐enabled design strategy for converting a depot‐forming phospholipid system into a surgical anti‐adhesion barrier. We show that replacing ethanol with propylene glycol strengthens hydration‐triggered gel reinforcement, prolongs intraperitoneal residence, maintains local immunomodulatory activity, and enables safer gram‐scale deployment across abdominal and tendon adhesion models.

## 2 Materials and Methods

### 2.1 Materials

Egg phosphatidylcholine (Lipoid E 80, phosphatidylcholine content > 80%) was obtained from Lipoid GmbH (Ludwigshafen, Germany). Medium‐chain triglyceride (MCT) was supplied by BASF (Ludwigshafen, Germany), and propylene glycol was purchased from Wonpoong Co., Ltd. (Seoul, Korea). 1,1'‐Dioctadecyl‐3,3,3',3'‐tetramethylindotricarbocyanine Iodide was purchased from Invitrogen (MA, USA). Isoflurane was purchased from Hana Pharmaceuticals Co., Ltd. (4004, Seoul, Korea). Quantitative real time PCR and cDNA kits were purchased from TAKARA Korea Biomedicals (RR430A and RR036A, Seoul, Korea). Primer sequences are listed in the Supporting Information.

### 2.2 Cell Lines

MEF and RAW264.7 cells were obtained from ATCC. The human pancreatic duct epithelial cell line (HPDE) was provided by Dr. Ming‐Sound Tsao. All cells were grown in medium at 37°C with 5% CO_2_. MEF and RAW264.7 cells were cultured in DMEM supplemented with 10% FBS. HPDE cells were grown in serum‐free medium supplemented with EGF and bovine pituitary extract.

### 2.3 Mice and Rats

The 5‐week‐old male C57BL/6 mice and 6‐week‐old male Sprague Dawley rats were housed at 3 per cage at 24°C under a 12‐h light/dark cycle. The mice and rats had access to chow diet (38057, Purina) and water ad libitum in a pathogen‐free facility, and their health status was monitored via culture, serum analysis, and microscopic examination. All experiments were conducted in accordance with relevant ethical regulations and guidelines at Korea National Institute of Health (BEC‐CAN‐2016‐001 and 002), and all studies were approved by the Institutional Animal Care and Use Committee of Yonsei University (IACUC‐A‐2025‐2001‐02 and IACUC‐A‐202504‐2031‐01).

### 2.4 Preparation of the Water‐Activated PPSG

PPSG was prepared by a slight modification of a reported protocol [31, 32, 33]. Egg phosphatidylcholine (Lipoid E 80) was dissolved in a 1:1 (w/w) mixture of medium‐chain triglyceride (MCT) oil and propylene glycol. A series of PPSGs containing 10–75 wt% egg phosphatidylcholine was prepared for initial screening (details in Supporting Information). The PPSG containing 50 and 75 wt% of egg phosphatidylcholine were denoted as PM50 and PM75, respectively. As a solvent‐control, EM75 was prepared by dissolving egg phosphatidylcholine (75 wt%) in a 1:1 (w/w) mixture of MCT oil and ethanol. Batches were made at 10 g scale. The mixtures were transferred to amber glass vials under nitrogen purging for 10 min and stirred overnight at 60°C and 250 rpm. After cooling to room temperature, formulations were stored at 4°C in amber vials until use.

### 2.5 Water‐Activated Gelation and Viscosity Measurements

Macroscopic gelation and viscosity changes upon water activation were evaluated for PM50, PM75, and EM75. Deionized water was added at 0, 1, 2, 5, 10, 15, 20, 30, and 50 wt% relative to the formulation mass, followed by spatula mixing for 10 s to induce shear‐assisted activation. Viscosity was measured at 200 rpm and room temperature using a rotational viscometer (DV2T‐LV, Brookfield, USA). Spindles LV‐61, LV‐62, or LV‐63 were selected as appropriate to maintain measurements within 10%–90% torque. All measurements were performed using three independently prepared batches per formulation (*n* = 3).

### 2.6 Oscillatory Rheology of PPSG

To compare gel properties with and without water activation, oscillatory rheology was performed using a rheometer (MCR 302e, Anton Paar, Austria) equipped with a 25‐mm parallel‐plate geometry (gap, 1.0 mm) at room temperature. For each formulation (PM50, PM75, and EM75), rheological measurements were conducted before and after water activation by adding deionized water (5 wt%) followed by mixing. Amplitude sweeps were conducted from 0.1% to 100% strain at a constant frequency of 10 Hz to obtain the storage modulus (G′) and loss modulus (G″). The linear viscoelastic (LVE) window was defined a priori (0.1%–0.5% strain), and the G′ plateau was summarized as the average G′ within the LVE. All measurements were performed using three independently prepared batches per formulation (*n* = 3).

### 2.7 Small‐Angle X‐Ray Scattering (SAXS)

Small‐angle x‐ray scattering (SAXS) measurements were performed using a Xeuss 2.0 system (XENOCS, France) with Cu Kα radiation (*λ* = 1.54189 Å). The sample‐to‐detector distance was set to 500 mm for PM50 (*q* = 0.001−0.70 Å^−^^1^) and 1000 mm for PM75 (*q* = 0.001−0.35 Å^−^^1^). Measurements were conducted at room temperature under vacuum with an exposure time of 1800 s. Because PM50 exhibits sol‐like behavior prior to water activation, whereas PM75 shows gel‐like behavior even before activation, different sample holders were used. Four conditions were examined: PM50 and PM75 without water, and PM50 and PM75 after activation with 5 wt% deionized water; activated samples were mounted immediately after mixing. PM50 samples were loaded into 1‐mm‐path‐length quartz capillaries, whereas PM75 samples were measured using a tape‐sandwich holder. All measurements were performed using three independently prepared batches per formulation (*n* = 3).

### 2.8 Adhesive Strength of PPSG

Adhesive strength was measured using a SurTa probe tack system (Chemilab, Republic of Korea). Guardix‐SG was equilibrated at 37.5°C before testing, whereas EM75 and PM75 were activated by adding 5 wt% PBS before testing. Each formulation (100 mg) was placed on fresh rat abdominal wall tissue and maintained at 37.5°C. A probe with a contact area of 1 cm^2^ was lowered at 0.05 mm s^−1^ and compressed onto the sample with a preload of 100 gf (0.981 N). The probe was then retracted at the same speed, and the maximum debonding force was recorded. Adhesive strength was calculated by normalizing the maximum debonding force to the probe contact area and expressed in pascals (*n* = 3).

### 2.9 Injectability Test of PPSG

Injectability was evaluated using a SurTa probe tack system (Chemilab, Republic of Korea) at room temperature. Guardix‐SG, EM75, and PM75 were loaded into a 3 mL syringe connected to a silicone tip with an inner diameter of 1 mm and a length of 1 cm. The syringe plunger was compressed by 10 mm at a speed of 1 mm s^−1^, followed by a holding time of 5 s after reaching the final position. The force generated during compression was recorded, and the maximum injection force was used for comparison (*n* = 3).

### 2.10 In Vitro Degradation Test

The in vitro degradation of EM75 and PM75 was evaluated by monitoring changes in residual gel mass over time. PPSG (0.5 g) was placed in a 2 mL microcentrifuge tube, followed by the addition of PBS (0.5 g). Samples were incubated in a shaking water bath at 37.5°C and 75 rpm. At each predetermined time point, the PBS phase containing the degraded fraction of PPSG was carefully removed, and the residual gel was weighed. Fresh PBS (0.5 g) was then added to each tube, and the samples were returned to the shaking water bath for continued incubation. The residual mass was calculated relative to the initial PPSG mass and expressed as a percentage (*n* = 3).

### 2.11 In Vivo Residence Study of PPSGs in Mice

Intraperitoneal residence of PPSG was quantified by near‐infrared fluorescence imaging. The oil phase of PPSG was labeled with 1,1′‐dioctadecyl‐3,3,3′,3′‐tetramethylindotricarbocyanine iodide (DiR; excitation 748 nm, emission 780 nm, Invitrogen, MA, USA) at 0.1 wt% relative to the formulation mass immediately before application. Male C57BL/6 mice aged 5 weeks (Raonbio, Republic of Korea) received 0.2 g of PPSG intraperitoneally. Imaging was performed on an IVIS spectrum (Perkin‐Elmer IVIS illumina) under isoflurane anesthesia at 0, 4, 8, 24, 48, 72, 120, and 168 h post‐application.

### 2.12 In Vivo Anti‐Adhesion Efficacy Study in a Peritoneal‐Cecum Adhesion Model

The anti‐adhesion efficacy of PPSG was evaluated in a peritoneal‐cecum adhesion rat model using adult male Sprague‐Dawley rats aged 6 weeks (Raonbio, Republic of Korea), randomly assigned to four groups (*n* = 10 per group): PBS (negative control), Guardix‐SG (clinical comparator), EM75 (ethanol‐MCT solvent control), and PM75. Under isoflurane anesthesia, injuries to the peritoneal wall and cecum were induced as previously described [34]. Next, 0.5 g of formulation was applied to fully cover the injured surfaces, after which the peritoneum and skin were closed with nylon sutures and the area was disinfected with povidone‐iodine. On postoperative Day 10, rats were euthanized and the abdomen re‐opened; adhesions between the injured surfaces were assessed by Zühlke classification for severity and extent of adhesion scoring (Table S1) [35].

### 2.13 In Vivo Anti‐Adhesion Efficacy Study in a Tendon Injury Model

The anti‐adhesion efficacy of PPSG was evaluated in a rat Achilles tendon injury model using adult male Sprague‐Dawley rats (6 weeks old; Raonbio, Republic of Korea). Animals were randomly assigned to three groups (*n* = 8 per group): PBS (negative control), Guardix‐SG (clinical comparator), and PM75. Under isoflurane anesthesia, the Achilles tendon was exposed and held under gentle tension using pins placed proximal and distal to the transection site to prevent tendon retraction. The tendon was then completely transected using a surgical blade and repaired using a modified Kessler suture technique with 6‐0 black silk (Ailee Co., Ltd, Republic of Korea). The skin was closed with 3‐0 black silk (Ailee Co., Ltd, Republic of Korea), and formulation (0.1 g) was then injected into peritendinous space using a 1 mL syringe. The surgical site was disinfected with povidone‐iodine. Two weeks after surgery, rats were euthanized by CO_2_ asphyxiation. The surgical site was re‐opened, and adhesion severity was scored according to Tang's classification (Table S1) [36].

### 2.14 Histological Analysis

Adhesion area tissues (injured parietal peritoneum‐cecum interface and Achilles tendon) and major organs (liver, heart, lung, spleen, and kidney) were harvested, fixed in 10% neutral buffered formalin for 48 h, paraffin‐embedded, and sectioned at 5 µm. Sections from adhesion sites were stained with hematoxylin and eosin (H&E) and Masson's trichrome. Major organs were stained with H&E for safety assessment. Slides were imaged using a slide scanner (OCUS40, Grundium) under identical acquisition settings; scale bars were embedded. Collagen area fraction (%) in Masson's trichrome‐stained sections was quantified using ImageJ.

### 2.15 Immunofluorescence Staining and Analysis and Immunoassays

RAW264.7 cells were seeded at 1×10^5^ cells per well on coverslips attached to 6‐well plates and incubated with DMEM media supplemented with 10% FBS. Cells were treated with LPS (100 ng/mL) and corresponding PPSG for 24 h. The cells on coverslips were fixed with ice cold methanol and washed with PBS 3 times. 1% BSA in PBS was used for blocking and cells were treated with anti‐CD206 (sc‐58986, 1:200) and anti‐CD80 (sc‐9091, 1:200) for 18 h. Alexa Fluor 488 goat anti‐mouse IgG and Alexa Fluor 594 goat anti‐rabbit IgG (1:500) were used as secondary antibodies. Nuclei were stained with DAPI (300 nM) for 5 min. After washing with PBS, cells were mounted and observed by confocal laser scanning microscopy (ZEISS). The media concentration of mouse IL‐6, IL‐1β, and TNF‐α were measured from the same cell sets using ELISA kits (R&D systems, #DY406, #DY401, and #DY410) according to the manufacturer's instructions.

### 2.16 Cell Lysis and Immunoblotting

Cells were washed once with DPBS and first lysed with lysis buffer (40 mM HEPES, pH 7.4, 0.5% Triton X‐100, 10 mM β‐glycerol phosphate, 10 mM pyrophosphate, and 2.5 mM MgCl_2_) with protease inhibitor (5 µg/mL aprotinin, 10 µg/mL leupeptin, and 250 µM PMSF) added. Then the lysed solution was sonicated on ice. Total protein concentration was measured by BCA Assay (Intron), and proteins were denatured in Laemmli sample buffer immediately before SDS‐PAGE. 20 µg of proteins was loaded to the SDS‐PAGE and transferred to PVDF membrane (Merck) via the Trans‐BlotR Turbo Blotting System (Bio‐Rad). Then the membranes were blocked in TBST buffer containing 2% BSA and incubated with respective primary antibodies for 12 h. The membranes were incubated with respective secondary antibodies (anti‐mouse or anti‐rabbit IgG conjugated with horseradish peroxidase, Invitrogen) for 1 h. Immunoblot signals were detected by MicroChemi (DNR Bioimaging system) with enhanced chemiluminescence, EzWestLumi (ATTO), and quantified by densitometry analysis of protein bands using DNR Bioimaging Tool. Immunoblot images are representative of three independent experiments.

### 2.17 RNAseq and Gene Enrichment Analysis

The quality of raw RNA‐seq data from each sample was assessed using FastQC (RRID:SCR_014583). TopHat (RRID:SCR_013035) was used to map reads from the RNA‐seq experiment to a reference genome (Rattus norvegicus Rnor_6.0). Then, the mapped reads were counted using HTSeq (RRID:SCR_005514) with the following parameters: htseq‐count ‐s no ‐m intersection‐nonempty ‐f bam. Differential gene expression analysis was done by the DESeq2 package (v. 1.24.0, RRID:SCR_000154) and manipulated the data in R (v3.6.1). Genes with low counts (<10) were filtered out and regularized log transformation was applied to minimize differences between samples with small counts. Gene set enrichment analysis (GSEA; RRID:SCR_003199) compared the difference between PBS treated and PM75 treated tissue. According to the Hallmark gene set, we divided subsets and calculated rlog FPKM FC values. Then, for each gene set, the mean of the rlog FPKM FC values of each sample was calculated.

### 2.18 CellTox Green‐Based Death Analysis

MEF, RAW264.7, and HPDE cells were seeded in 96‐well plates and incubated for 24 h. After treating the cells with 0.1 g of PPSG, the cell culture medium was exchanged with each corresponding medium containing CellTox Green Dye (Promega, #G8741). Phase and green fluorescence images were acquired by IncuCyte Zoom. IncuCyte Zoom basic analyzer was utilized to perform quantitative analysis. The presence of green color (CellTox object count/mm^2^ over time) was obtained to quantify the number of dead cells.

### 2.19 Hemolysis Ratio

Fresh blood was extracted from each animal and stabilized with heparin. 2 mL of whole blood same was added to PBS and then centrifuged at 10 000 × g for 5 min. Red blood cells were diluted with 20 mL of PBS. 0.2 mL of the diluted red blood cells were then exposed to each PPSG along with distilled water and normal saline as control groups. After incubation at room temperature for 4 h, each tube was centrifuged at 10 000 × g for 5 min. 0.1 mL of the supernatant of all samples were sampled to a 96‐well plate and the absorbance was measured with a microplate reader (Infinite 200 PRO, Tecan) at 570 nm.

### 2.20 Gene Expression Analysis

Total RNA was extracted from the tissues using the MiniBEST Universal RNA Extraction Kit (TAKARA) following the supplier's protocol. For cDNA synthesis, 1 µg of RNA was reverse transcribed with the PrimeScript first Strand cDNA Synthesis Kit (TAKARA). The reaction was carried out at 42°C for 45 min, after which the enzyme was heat‐inactivated at 90°C for 5 min. For quantitative real time PCR, the cDNA was diluted 1:4 with nuclease‐free water, and amplification was performed on a StepOnePlus Real‐Time PCR system (Applied Biosystems). Expression levels were normalized to GAPDH.

### 2.21 Serum Profiles for Biomarkers of Liver and Renal Injury

0, 4, 8, 24, 48, and 120 h after PPSG administration, blood was collected from each animal, and serum was obtained by centrifugation at 5000 × g for 20 min. The concentrations of aspartate aminotransferase (AST), alanine aminotransferase (ALT), and blood urea nitrogen (BUN) were measured using commercial assay kits for AST (BioAssay Systems, EASTR‐100), ALT (BioAssay Systems, EALT‐100), and BUN (BioAssay Systems, DIUR‐100) in accordance with the manufacturers’ instructions.

### 2.22 Flow Cytometry Analysis of CD80 and CD206

RAW264.7 cells were seeded at 1×10^6^ cells and incubated with DMEM media supplemented with 10% FBS. Cells were added with LPS (100ng/mL) and corresponding PPSG for 24 h. Cells were detached with versene and collected through centrifugation at 900 x g for 5 min. Cells were stained with anti‐mouse PE‐Cy7 F4/80 (123113, Biolegend, 1:100), PE CD206 (141705, Biolegend, 1:100), and FITC CD80 (104705, Biolgened, 1:100) for 18 h. Live and dead cells were excluded via viability stain (AB_2869405, BD Biosciences). Finally, the samples were collected and analyzed via flow cytometry (BD FACSAria III) and Flow Jo (BD Biosciences). Briefly, singlets were identified based on FSC‐A versus FSC‐H, and live cells were gated using a viability dye. From the live‐cell population, macrophages were defined as F4/80^+^ cells, with gates set using the corresponding unstained/FMO controls. F4/80^+^ macrophages were then further subdivided into CD80^+^ or CD206^+^ subsets, with thresholds similarly determined using unstained/FMO controls.

### 2.23 Statistics and Reproducibility

All statistical analyses were performed using GraphPad Prism 7. Rheology data were analyzed using one‐way ANOVA with Sidak's multiple‐comparisons post hoc test, and data are presented as mean ± SD. IVIS data (mouse studies) were analyzed using unpaired two‐tailed Student's *t*‐test, and data are presented as mean ± SD. Adhesion strength, injection force, histology and qRT‐PCR data were analyzed using one‐way ANOVA with Tukey's multiple‐comparisons post hoc test, and data are presented as mean ± SD. Adhesion scoring data (rat studies) were analyzed using the Kruskal–Wallis test followed by Dunn's multiple‐comparisons post hoc test, and data are presented as mean ± SE.

For formulation‐based experiments, measurements were performed using independently prepared batches as indicated. Animals (mice for IVIS studies and rats for adhesion models) were randomized into groups and treated according to the experimental design. No statistical methods were used to predetermine sample sizes; however, group sizes were chosen based on prior similar studies in the field. No data were excluded from the analyses.

## 3 Results

### 3.1 Mechanical Characterization of PPSG

To delineate the critical material parameters required for surgical barrier conversion, we systematically screened candidate PPSG compositions for hydration‐triggered mechanical reinforcement (Figure S1A–E). Based on this initial screen, formulations containing 75 wt% PC were selected for a direct comparison between PM75 (propylene glycol‐based) and EM75 (ethanol‐based). PM50 was also included as a reference to illustrate the water‐activated sol–gel transition characteristic of PPSG.

Macroscopic appearance of the formulations evolved distinctly with increasing water content (0, 5, and 30 wt%; Figure 2A), revealing a clear hydration‐dependent sol–gel transition. Specifically, PM50 readily underwent gel formation at 5% water, whereas EM75 remained in its low viscosity sol state at 5% and transitioned into gel state only at water content of 30%. Notably, PM75 exhibited gel state even in anhydrous state which was further reinforced upon hydration. Consistent with these observations, rotational viscosity of both PM50 and PM75 increased sharply upon the addition of 5% water, while EM75 exhibited negligible changes at this at 5% water and required higher water contents to develop appreciable gel character (Figure 2B). PM75 achieved a 1.41‐fold higher peak viscosity than EM75 and maintained a smaller post‐peak decrease profile, indicating greater water sensitivity and mechanical robustness.

**FIGURE 2:**
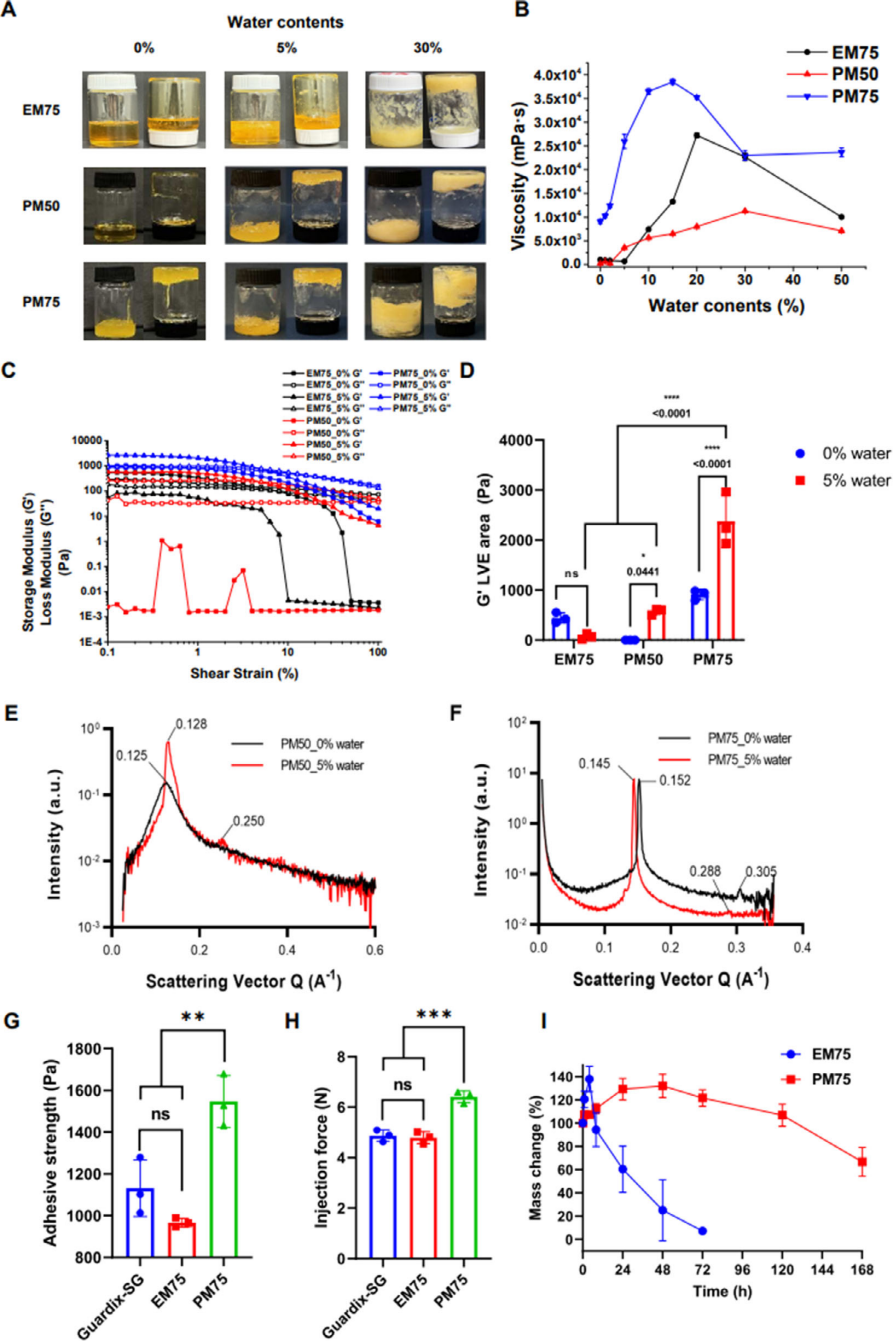
Mechanical characterization of PPSGs. (A) Representative inverted‐vial images of PPSGs at 0%, 5%, and 30% added water, showing water‐dependent gelation. (B) Viscosity profile of PPSGs as a function of water content (1%–50%) (*n* = 3). (C) Rheological properties of PPSGs from amplitude sweep tests at 10 Hz (*n* = 3). (D) The storage modulus (G′) of PPSGs in the linear viscoelastic region (*n* = 3). (E, F) Small‐angle x‐ray scattering profiles of PPSGs before and after activation with 5% water: (E) PM50 and (F) PM75. (G) Adhesive strength of PPSGs and Guardix‐SG measured on rat abdominal wall tissue under wet conditions (*n* = 3). (H) Maximum injection force of PPSGs and Guardix‐SG measured using a syringe‐based injection test (*n* = 3). (I) In vitro degradation profiles of EM75 and PM75, determined from changes in residual gel mass over time (*n* = 3). Statistical significance in (D) was determined by one‐way ANOVA with Sidak's multiple‐comparisons post hoc test. Statistical significance in (G, H) was determined by one‐way ANOVA with Tukey's multiple‐comparisons post hoc test. ****p < 0.0001, ***p < 0.001, and *p < 0.05.

Oscillatory rheology further highlighted these divergent viscoelastic properties. In amplitude sweep tests (0.1%–100% strain at 10 Hz), EM75 exhibited a liquid‐like response at 5% water content, with storage modulus (G′) remaining below the loss modulus (G″). In contrast, PM50 displayed a gel‐like response after the addition of 5% water (G′ > G″), while PM75 consistently demonstrated gel‐like features even in anhydrous state (G′ > G″) that were further reinforced upon the addition of 5% water (G′ >> G″) (Figure 2C). In alignment with these trends, hydration of 5% water significantly elevated the G′ plateau within the linear viscoelastic (LVE) region for both PM50 and PM75 (*p* = 0.0441 and *p* < 0.0001, respectively), whereas EM75 showed no significant alteration. After activation, the LVE‐region G′ of PM75 was higher than that of PM50 and EM75 by 4.14‐fold and 33.65‐fold, respectively (*p* < 0.0001; Figure 2D). Together, these data indicate that water activation markedly strengthens PPSG mechanics with PM75 providing the highest gel structural robustness among the tested formulations.

To further investigate gel microstructure, small‐angle x‐ray scattering (SAXS) was performed (Figure 2E,F). PM50 exhibited a single peak at 0.125 Å^−1^ in the absence of water; after 5% hydration, secondary maxima at ≈2q* (0.128 → 0.250 Å^−1^), indicating the development of lamellar stacking. PM75 also displayed secondary maxima at ≈2q* (0.152 → 0.305 Å^−1^) even without water, which persisted after hydration (0.145 → 0.288 Å^−1^), indicating tighter lamellar stacking upon water activation. To evaluate translational barrier utility and tissue adhesive strength under physiological conditions, PPSG formulations were compared with the clinical comparator, Guardix‐SG, using fresh rat abdominal wall tissue (Figure 2G). Guardix‐SG, which undergoes thermosensitive gelation at 37.5°C, showed an adhesive strength of 1131.3 ± 135.5 Pa. While EM75 activated with 5 wt% PBS exhibited a non‐significant difference in the adhesive strength of 966.5 ± 21.0 Pa compared to Guardix‐SG (*p* = 0.2223), PM75 displayed a significantly superior adhesive strength of 1546.7 ± 124.6 Pa compared with both Guardix‐SG and EM75 (*p* = 0.0075 and *p* = 0.0014, respectively).

Subsequently, injectability of the formulations was evaluated by measuring the maximum injection force (Figure 2H). Guardix‐SG and EM75 showed comparable injection forces of 4.87 ± 0.23 N and 4.79 ± 0.24 N, respectively, with no significant difference between the two groups (*p* = 0.9080). Due to its highly reinforced internal matrix, PM75 required a higher maximum injection force of 6.41 ± 0.24 N, which was significantly greater than those of Guardix‐SG and EM75 (*p* = 0.0005 and *p* = 0.0004, respectively).

The in vitro degradation profiles of EM75 and PM75 were evaluated by monitoring changes in residual gel mass over time (Figure 2I). Reflecting its sol‐like property, EM75 underwent rapid initial swelling, peaking at a residual mass of 137.9% ± 10.9% at 4 h, followed by rapid degradation that led to near‐complete loss of matrix integrity by 72 h. In contrast, PM75 reached a maximum residual mass of 132.1% ± 10.0% and exhibited a sustained and controlled erosion profile that extended over 168 h, validating its capacity for prolonged barrier retention. Collectively, PM75 exhibited superior water‐induced mechanical reinforcement and the greatest adhesive strength, while remaining injectable under the tested syringe condition and showing prolonged in vitro matrix retention. Therefore, PM75 was designated as the candidate PPSG formulation for subsequent in vitro and in vivo validation studies, with EM75 as the solvent control.

### 3.2 In Vivo Residence Study of PPSG

To evaluate in vivo residence kinetics of the developed formulations, we performed non‐invasive near‐infrared IVIS imaging using DiR‐loaded PPSG formulations (Figure 3A–C). The progressive reduction in fluorescence intensity at the surgical application site was monitored as a surrogate metric for the in vivo degradation and clearance of the PPSG barriers. High intensity fluorescence signals at 0 h confirmed the uniform and successful application of all formulations at the intra‐abdominal surgical site (Figure 3A). By Day 7, local fluorescence dropped below the detection limit across all groups, confirming that these transient phospholipid barriers underwent complete clearance in vivo. Notably, the degradation kinetics differed between the two formulations. The ethanol‐based EM75 remained largely intact for approximately 48 h, but underwent rapid structural collapse and clearance by 72 h, indicating limited stability in the peritoneal cavity (Figure 3B). In contrast, propylene glycol‐based PM75 displayed substantially prolonged retention, with its fluorescence signal declining gradually over the course of 1 week, validating its improved residence within the peritoneal cavity (Figure 3B). Quantitative integration of these normalized fluorescence‐time profiles via area under the curve (AUC) analysis further substantiated these structural differences (Figure 3C), with PM75 exhibiting a significantly higher AUC than EM75 (*p* = 0.0151). Collectively, these results establish that substituting the solvent system with propylene glycol optimizes the physical barrier framework, enabling it to withstand physiological clearance mechanisms and maintain the sustained in vivo residence required to guide successful tissue remodeling.

**FIGURE 3:**
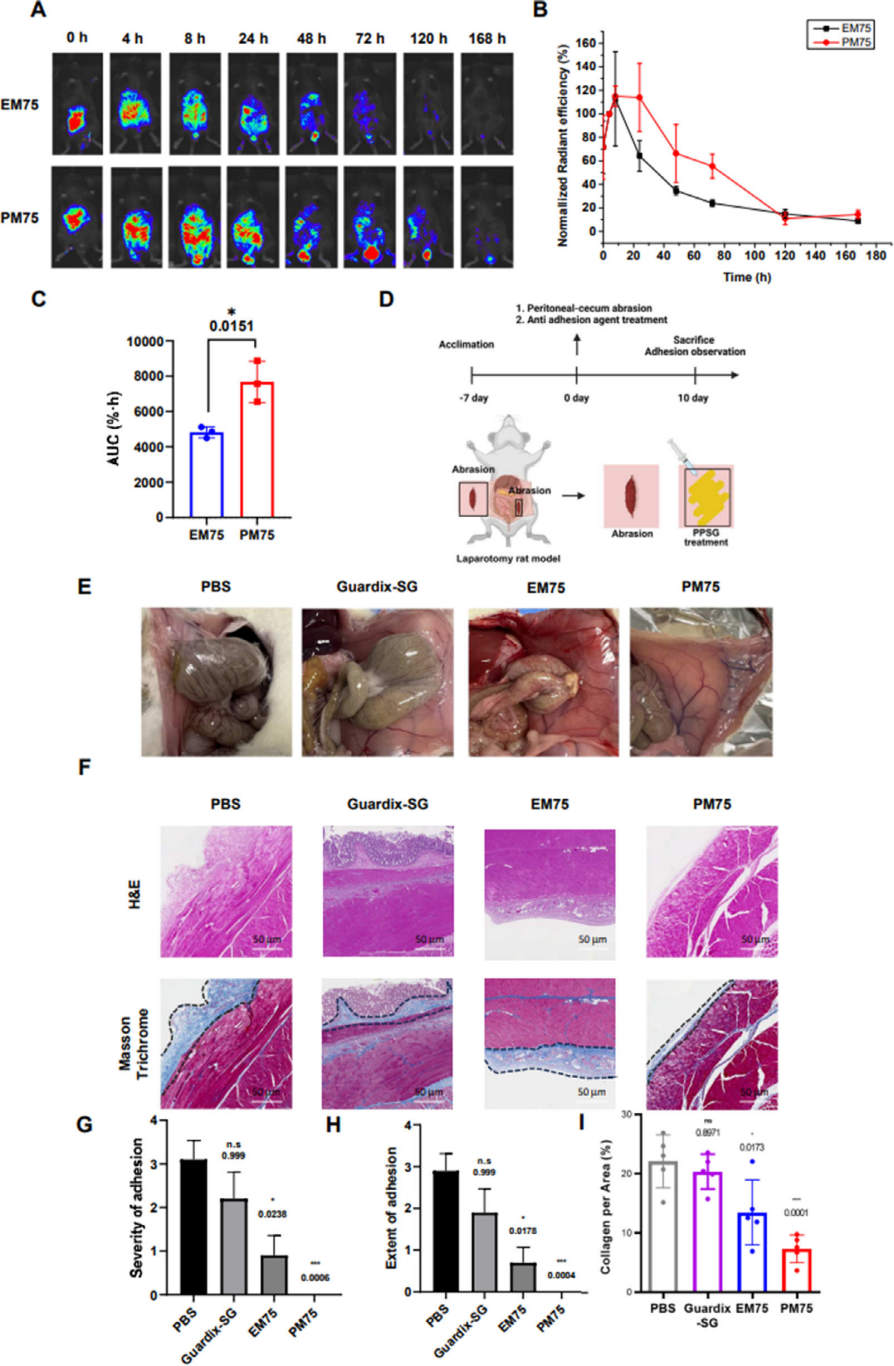
In vivo residence profile and therapeutic efficacy evaluation of PPSGs. (A) Representative IVIS images of DiR‐loaded PPSGs acquired at predetermined time points. (B) In vivo residence profiles based on normalized fluorescence intensity (*n* = 3). (C) Quantification of area under the curve (AUC) of normalized fluorescence intensity over 7 days (*n* = 3). (D) Schematic illustrations of rat peritoneal‐cecum adhesion model and PPSG treatment. (E) Representative gross images showing adhesion prevention by PPSGs at Day 10 post‐surgery. (F) Representative H&E and Masson's trichrome staining images of adhesion sites on Day 10 post‐surgery. (G, H) Quantification of adhesion scores for (G) severity and (H) area at Day 10 post‐operation (*n* = 10). (I) Quantification of collagen deposition area (%) (*n* = 5). Data are shown as mean ± SD (C, I) or mean ± SE (G, H). Statistical analysis: unpaired two‐tailed Student's *t*‐test (C), one‐way ANOVA with Tukey's multiple‐comparisons post hoc test (I); Kruskal–Wallis test followed by Dunn's multiple‐comparisons post hoc test (G, H). *p < 0.05, **p < 0.01, ***p < 0.001, and ****p < 0.0001.

### 3.3 In Vivo Anti‐Adhesion Efficacy Study in a Peritoneal‐Cecum Adhesion Model

To determine the functional therapeutic capacity of the developed formulations, we evaluated their anti‐adhesion efficacy in a robust rat peritoneal‐cecum abrasion model (Figure 3D). After inducing standardized injuries on Day 0, formulations were applied to ensure comprehensive coverage of the injured surfaces, and the terminal adhesion outcomes were assessed on postoperative Day 10. Experimental groups included PBS (no‐treatment control), Guardix‐SG (clinical comparator), ethanol‐based PPSG (EM75), and propylene glycol‐based PPSG (PM75). Gross macroscopic evaluation revealed dense and severe adhesions in the PBS and Guardix‐SG groups, whereas EM75 mitigated overall adhesion formation and PM75 prevented macroscopic adhesion development across all treated animals (Figure 3E). Formulations with lower phosphatidylcholine content, including EM50 and PM10–50 series, revealed a higher incidence of adhesion formation than their EM75 and PM75 counterparts (Figure S2A–C). Histological assessment strongly corroborated these findings (Figure S3). H&E and Masson's trichrome staining revealed dense collagen deposition at adhesion sites in PBS‐ and Guardix‐SG‐treated rats, whereas local collagen deposition was reduced in the EM75 and PM75 groups (Figures 3F and S4). Quantification of the collagen area fraction (%) confirmed significant reductions in the fibrotic tissue area for both EM75 and PM75 compared with PBS (*p* = 0.0173 and *p* < 0.0001, respectively) (Figure 3I). To systematically assess adhesion severity and its extent, standardized 0–4 ordinal grading scale was applied for quantification (Figure 3G,H and Table S1). Given the ordinal and discrete nature of these clinical scoring parameters across animals, inter‐group comparisons were performed using the Kruskal‐Wallis test followed by Dunn's multiple comparisons. In the PBS group, 90% of the animals manifested extensive and severe peritoneal‐cecum adhesions that required sharp dissection for physical separation, resulting in high severity and extent scores. In the Guardix‐SG group, adhesions occurred in 60% of the animals, and severity and extent scores among affected subjects did not significantly deviate from the untreated control PBS group. The administration of EM75 limited adhesions to only 30%; both adhesion severity and extent were significantly reduced (*p* = 0.0238 and *p* = 0.0178, respectively). In contrast, the PM75 group exhibited complete absence of adhesions, with significant reduction in the severity and extent scores (*p* = 0.0006 and *p* = 0.0004, respectively). Notably, PM75 elicited a uniform and reproducible therapeutic outcome across all animals, whereas EM75 left residual and persistent adhesions in a subset of the treated subjects, highlighting the therapeutic superiority of the propylene glycol formulation.

### 3.4 PPSG Induces Immunomodulation to Inhibit Fibrosis

Given that phosphatidylcholine has been associated with anti‐inflammatory effects [23, 24, 25], we sought to examine whether these activities remain functionally coupled to a structured gel barrier in the dynamic post‐surgical environment. To unravel the physiological mechanisms driving the anti‐adhesive performance of the PPSG platform, we comprehensively interrogated its local immunomodulatory effect. We tested the effects of PBS, EM75, and PM75 on LPS activated RAW264.7 mouse macrophage cell line for 24 h (Figure 4A,B). Following classical LPS activation and treatment with 0.1 g of PBS, EM75, and PM75, immunofluorescence analysis was performed for immune population analysis of CD80 (M1 macrophage marker) and CD206 (M2 macrophage marker). Since M1 macrophages orchestrate the downstream inflammation‐driven fibrosis following tissue injury, whereas M2 macrophages exert anti‐inflammatory effects that counteract M1 induced fibrotic transitions, we reasoned that PPSG‐mediated anti‐adhesion is intrinsically linked to macrophage polarization. As anticipated, baseline LPS stimulation elevated CD80 expression in control RAW264.7 cells. In alignment with in vivo results, both EM75 and PM75 markedly reduced CD80 expression while enhancing CD206 expression (Figure 4A), driving a robust phenotypic shift toward an anti‐inflammatory phenotype despite sustained inflammatory LPS stimulation. This effect was further validated by flow cytometry analysis of F4/80‐positive RAW264.7 cells stained for CD80 and CD206 (Figure 4B). The CD206‐positive population increased to 66.9% and 63.1% with EM75 and PM75 treatment, respectively, compared to 8.10% with PBS (Figure 4B). Conversely, the CD80‐positive macrophage population decreased concomitantly from 76.9% with PBS to 33.0% and 15.0% for EM75 and PM75 treatment, respectively, confirming a strong immunomodulatory effect by PPSG (Figure 4B). Collectively, these data demonstrate that PPSG drives macrophage polarization toward the anti‐inflammatory M2 phenotype, thereby suppressing inflammation and providing a mechanistic basis for its anti‐adhesive properties. To further substantiate this immunosuppressive phenotype, we quantified the transcription profiles with mRNA expression levels of key pro‐inflammatory cytokines (TNF‐α, IL‐6, and IL‐1β) in LPS stimulated RAW264.7 cells (Figure 4C‐E). While baseline un‐stimulated macrophage parameters remained statistically unaltered across the groups, the transcript levels of all three cytokines were significantly reduced upon treatment with either EM75 or PM75. Importantly, ELISA confirmed this anti‐inflammatory effect at the protein level, revealing significantly reduced secretion of TNF‐α, IL‐6, and IL‐1β following exposure to EM75 and PM75.

**FIGURE 4:**
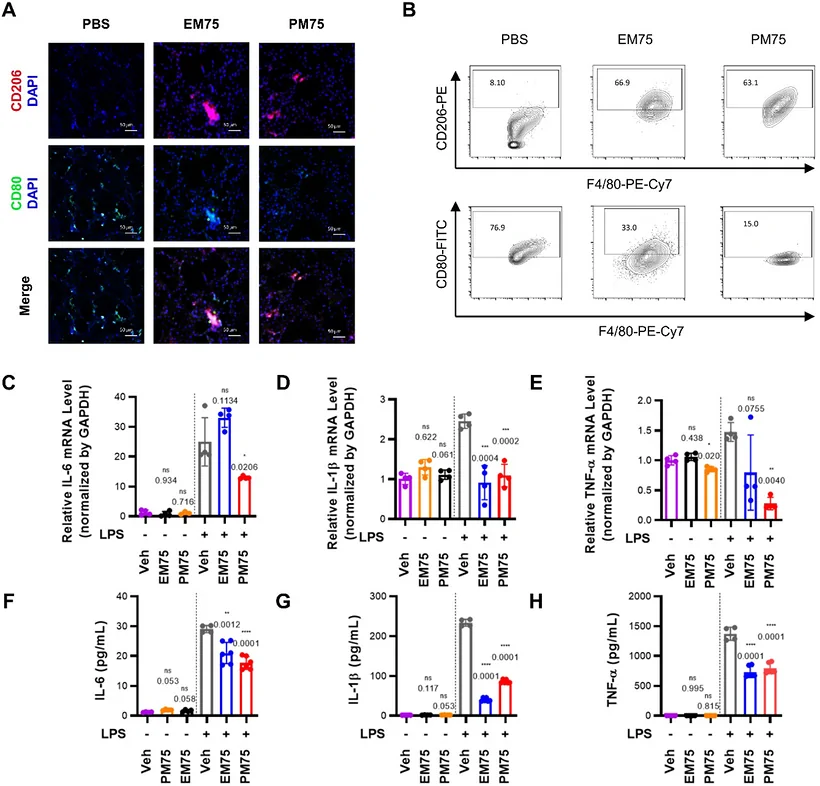
PPSG induces immunomodulation in vitro. (A) Representative CD80 and CD206 immunofluorescence staining of LPS‐treated RAW264.7 cells incubated with PBS, EM75, and PM75. RAW264.7 cells were treated with LPS (10 ng/mL) with 0.1 g of PBS, EM75, or PM75 for 24 h and subjected to fixation and staining with respective antibodies for 18 h. (B) Flow cytometry analysis of LPS‐treated F4/80‐positive RAW264.7 cells. Population gated as CD206‐positive (top) or CD80‐positive (bottom) are represented. (C–E) mRNA expression of inflammatory cytokines IL‐6 (C), IL‐1β (D), and TNF‐α (E) measured by qRT‐PCR are shown (*n* = 4, biological replicates). (F–H) Protein level of inflammatory cytokines IL‐6 (F), IL‐1β (G), and TNF‐α (H) measured by ELISA are shown (*n* = 4 or 6, biological replicates). All the data are presented as the mean ± SD and analyzed by one‐way ANOVA with Tukey's multiple‐comparisons post hoc test. ****p < 0.0001, ***p < 0.001, **p < 0.01, and *p < 0.05 (C–H).

To define the precise cellular and molecular signaling circuits driving PPSG mediated anti‐inflammatory response that contributes to its anti‐adhesive effects, we conducted whole transcriptome analysis using RNA extracted from rat peritoneal‐cecum tissues collected from the in vivo study (Figure 5A). Hallmark gene set enrichment analysis (GSEA) of differentially expressed genes (DEG) revealed that seven of the top nine enriched pathways were fundamentally associated with suppressed immune and inflammatory response (Figure 5B). Notably, the most significantly enriched pathway was TNF‐α signaling via NF‐κB, presenting a strong molecular rationale that PM75 mediates immunomodulation by inhibiting the classical NF‐κB activation axis. To test this hypothesis, we assessed the phosphorylation status of NF‐κB pathway axis in LPS‐stimulated RAW264.7 macrophages via western blotting (Figure 5C). PM75 treatment reduced levels of activated NF‐κB, which was further supported by a decrease in phosphorylated IκBα (Figure 5C). However, pharmacological activation of this signaling axis via NF‐κB activator, Compound 32 (Cmpd 32), restored NF‐κB activation and blunted the PM75‐induced immunomodulatory phenotype (Figure 5C). Collectively, these mechanistic insights confirm that anti‐adhesive capacity of the PM75 barrier is molecularly supported by a targeted inhibition of NF‐κB signaling axis.

**FIGURE 5:**
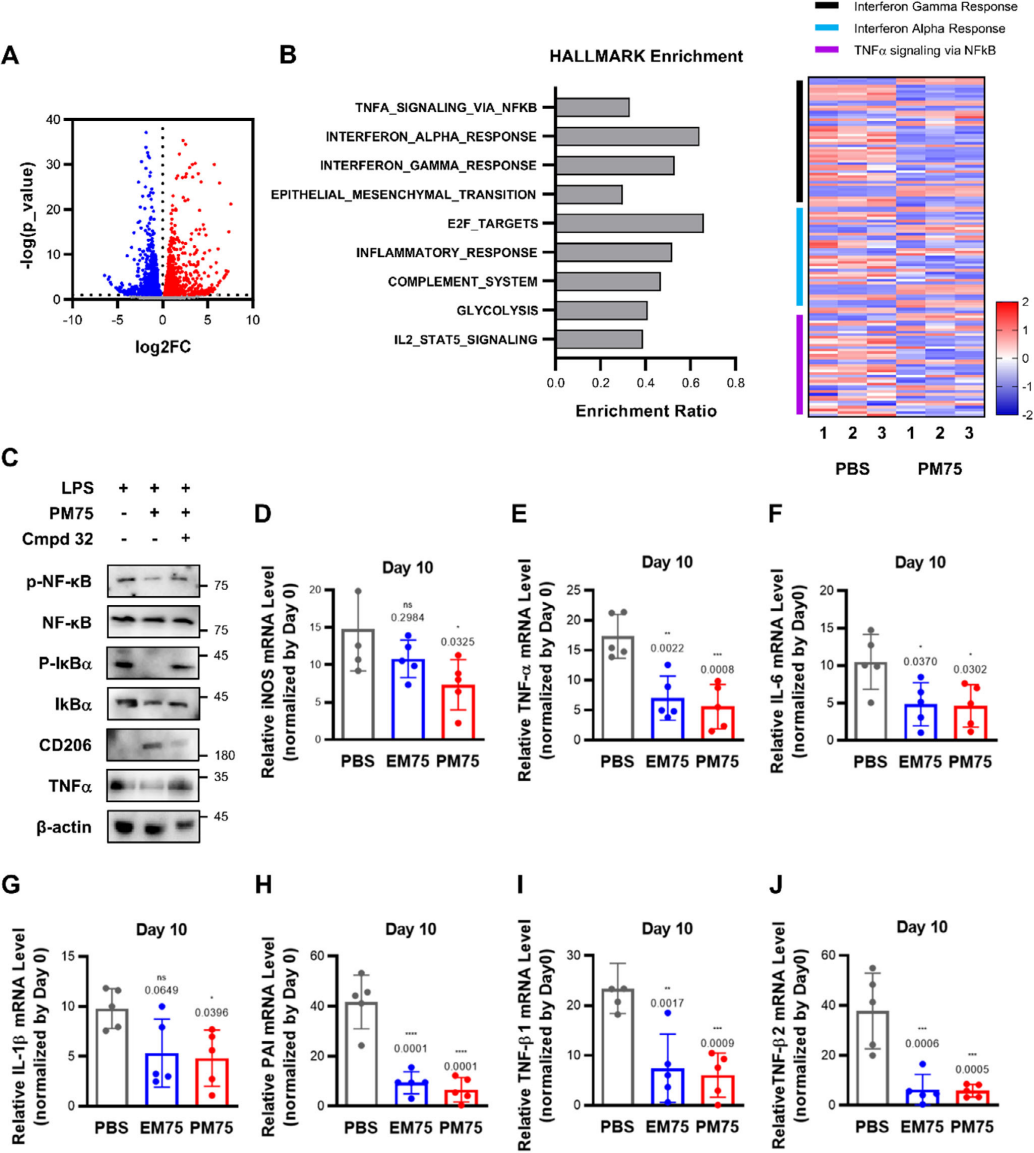
PM75 suppresses NF‐κB signaling to attenuate inflammatory and fibrotic responses. (A) Volcano plot of total mRNA sequencing data of PBS‐ and PM75‐treated rat peritoneal‐cecum adhesion tissue. (B) (Left) Hallmark gene set enrichment analysis (GSEA) of the differentially expressed genes from the mRNA sequencing data. (Right) Heatmap of enriched gene set of interferon gamma response, interferon alpha response, and TNF‐α signaling via NF‐κB showing decreased expression in PM75 compared to PBS. (C) Western Blotting analysis of protein expression of NF‐κB signaling pathway and macrophage markers of RAW264.7 cells pre‐incubated with LPS (10ng/mL) for 24 h and treated with PBS, PM75, or PM75 + Compound 32 (NF‐κB activator) for 24 h. (D–J) mRNA expression levels at Day 10 normalized to Day 0 levels of animal tissues on the region of surgery treated with PBS, EM75, or PM75 (*n* = 5). Inflammatory cytokine levels of iNOS (D), TNF‐α (E), IL‐6 (F), and IL‐1β (G) are shown. Fibrosis pathway related genes of PAI (H), TGF‐β1 (I), TGF‐β2 (J) are shown. All the data are presented as the mean ± SD and analyzed by one‐way ANOVA with Tukey's multiple‐comparisons post hoc test. ****p < 0.0001, ***p < 0.001, **p < 0.01, and *p < 0.05 (C–J).

To validate whether this anti‐inflammatory axis successfully operates in vivo, animals subjected to anti‐adhesion therapy with 0.5 g of PBS, EM75, or PM75 were sacrificed on Day 2 and Day 10 post‐surgery to monitor localized gene expression dynamics at the surgical boundaries. At the acute Day 2 checkpoint, expression of pro‐inflammatory cytokines (iNOS, TNF‐α, IL‐6, and IL‐1β) were uniformly increased and showed no significant differences between PBS‐ and PPSG‐treated groups (Figure S5A–D). This transient, acute inflammatory spike represents the expected physiological response to mechanical surgical trauma. By Day 10, however, expression of iNOS, TNF‐α, IL‐6, and IL‐1β were markedly reduced in EM75‐ and PM75‐treated animals (Figure 5D‐G), indicating sustained immunomodulatory effects in vivo aligning with in vitro findings. To determine whether this targeted suppression of chronic inflammation was accompanied by reduced fibrosis, fibrotic gene expression (PAI, TGF‐β1, and TGF‐β2) was assessed. While these fibrotic transcripts remained largely unaltered during the acute Day 2 checkpoint, except for a minor acute anti‐fibrotic effect of the EM75 treated group (Figure S5E–G), EM75‐ and PM75‐treated animals exhibited markedly reduced expression of these fibrotic genes by Day 10 (Figure 5H–J), consistent with inhibition of fibrosis and reduced tissue adhesion (Figure 4E–H). Overall, these findings clarify that PPSG, particularly EM75 and PM75, do not function merely as passive physical barriers, but also act as an immunomodulatory agent that suppresses inflammation and fibrosis, thereby contributing to its anti‐adhesive properties in vivo.

### 3.5 Biocompatibility of PPSG In Vitro and In Vivo

While unique physicochemical properties of the PPSG enable it to conformally coat injured tissue boundaries, establishing a robust physical barrier while simultaneously driving anti‐fibrotic immunomodulation, comprehensive biocompatibility remains a strict prerequisite for clinical translation. To systematically address this requirement, EM75 and PM75, the most effective PPSG formulations were subjected to rigorous in vitro and in vivo biocompatibility evaluations. First, human pancreatic epithelial cells, mouse fibroblasts, and mouse macrophages were exposed to 0.1 g of either EM75 or PM75 (Figure 6A–C). CellTox Green analysis revealed that EM75 induced moderate cytotoxicity to mouse fibroblasts and mouse macrophages, whereas PM75 exerted no detectable toxicity and did not affect cell growth. Despite the observed low cytotoxicity of PM75, further in vivo assessment was performed to scrutinize tissue‐level and systemic compatibility. Histological analysis of liver, heart, lung, spleen, and kidney were performed in animals treated with anti‐adhesion therapy with 0.5 g of PBS, EM50, EM75, PM10, PM25, PM50, and PM75 (Figures 6D and S6A–E). Livers from EM75‐treated animals displayed mild increase in lymphocyte infiltrates, indicative of a focal inflammatory response within cavitary lesions. In contrast, no significant histopathological alterations were detected across the other vital organs examined. Importantly, PM75 did not provoke any detectable structural or histopathological damage in the liver or other organs. To examine potential hemolytic toxicity, hemolysis ratios were measured (Figure S7A). Propylene glycol‐based PPSG formulations, including PM75, showed negligible hemolysis. In contrast, EM50 manifested an elevated hemolysis ratio, which is likely attributable to its ethanol content damaging the cellular membrane [37]. However, in line with this mechanism, as ethanol content decreased, the hemolysis ratio of EM75 decreased significantly. Systemic biocompatibility and metabolic organ function was further tracked by measuring acute and post‐operative levels of AST, ALT, and BUN in animals treated with 0.5 g of PBS, EM75, PM75 or Guardix‐SG (Figure 6E,F). Animals receiving EM75 treatment significantly elevated liver toxicity markers (AST and ALT) both acutely (up to Day 2) and post‐operatively (Day 10), which may be a marker for mild increase in lymphocyte infiltrates in the liver histology (Figure 6D). Upon increase of ethanol content, EM50 showed marked increase in the toxicity panel consistent with the histology data (Figure S7B,C). In contrast, the administration of PM75 induced no deviations in AST, ALT, or BUN levels (Figure 6E,F). Collectively, these safety profiles demonstrate that while ethanol‐based PPSG formulations such as EM50 exhibit cytotoxicity and liver toxicity at this scale, propylene glycol‐based PPSG, particularly PM75, achieves exceptional systemic and tissue‐level biocompatibility and safety in vitro and in vivo. This highlights its clinical potential as a safe post‐operative anti‐adhesion agent.

**FIGURE 6:**
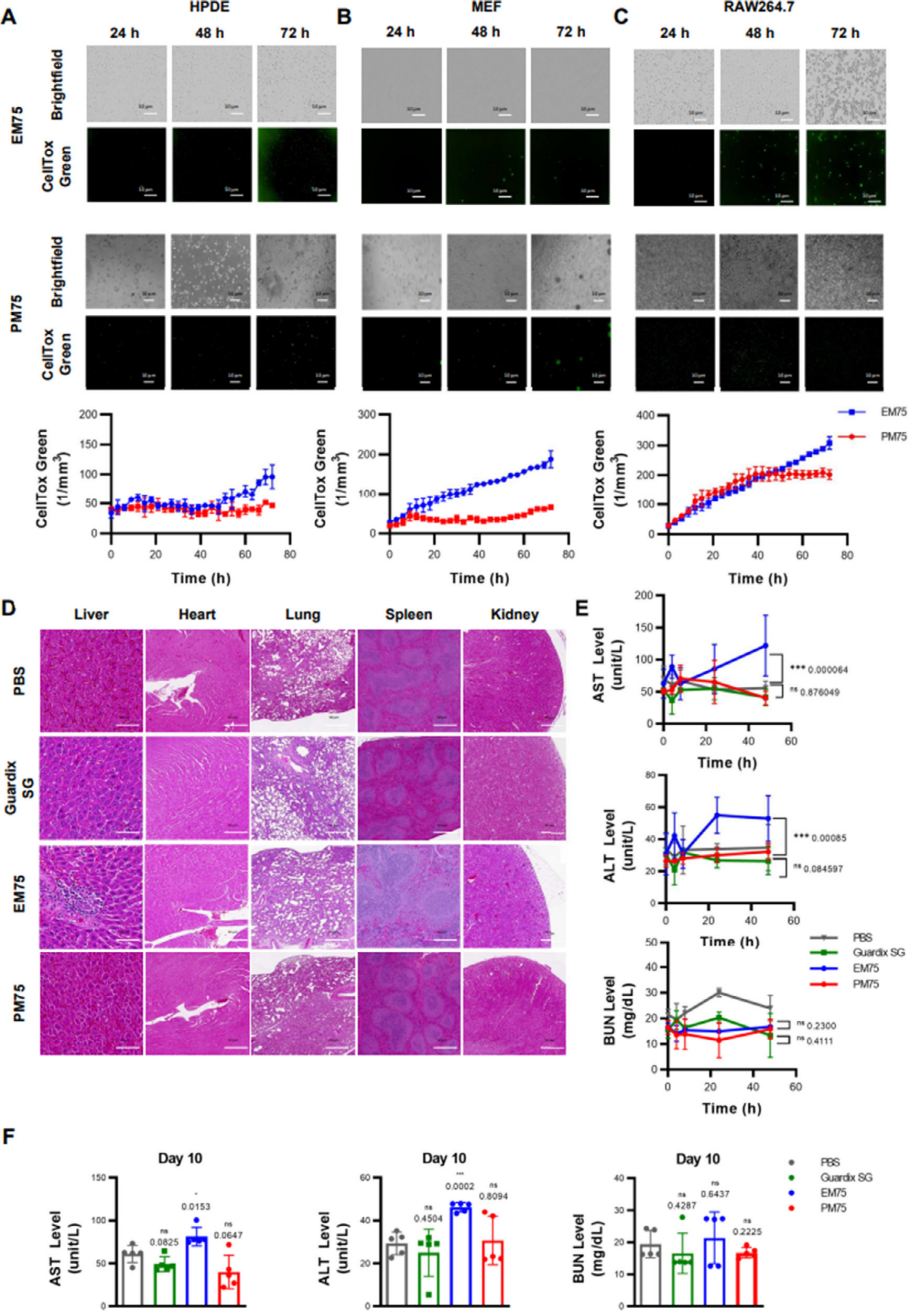
Biocompatibility of PPSG in vitro and in vivo. (A–C) CellTox Green analysis of dead cells of HPDE (A), MEF (B), and RAW264.7 (C), treated with EM75 and PM75 for 72 h. Representative brightfield and CellTox Green images of cells treated with EM75 (top) and PM75 (middle) at 24, 48, and 72 h. Quantitative graph of the amount of death cells are represented (bottom). (D) H&E staining of liver, heart, lung, spleen, and kidney of mouse treated with PBS, Guardix‐SG, EM75, and PM75 at Day 10. Liver histology of EM75 displays lymphocyte infiltration representing inflammation. (E, F) Blood levels of AST, ALT, and BUN were measured to assess the acute (*n* = 10) (E) and post‐operative (*n* = 5) (F) toxicity of PBS, Guardix‐SG, EM75, and PM75. All the data are presented as the mean ± SD. All *p* values were determined by Student's unpaired two‐tailed *t*‐test (E) and one‐way ANOVA with Tukey's multiple‐comparisons post hoc test (F). ****p < 0.0001, ***p < 0.001, **p < 0.01, and *p < 0.05 (E, F).

### 3.6 In Vivo Anti‐Adhesion Efficacy Study in a Tendon Injury Model

To demonstrate the translational potential of the developed agents across anatomically diverse surgical settings, we evaluated their anti‐adhesion efficacy in a rat Achilles tendon injury model (Figure 7A). After tendon immobilization, complete surgical transection, and subsequent structural repair, peritendinous adhesions were assessed during wound healing. Groups comprised PBS, Guardix‐SG, and PM75. Gross macroscopic inspection after 2 weeks post‐surgery revealed the development of severe and extensive adhesions in the PBS group, whereas both Guardix‐SG and PM75 reduced adhesion formation (Figure 7B). To quantify these treatment outcomes, peritendinous adhesion severity was characterized and scored using a standardized 0–4 ordinal scoring scale (Figure 7C and Table S1). In PBS treated rats, dense and extensive fibrous adhesions compromised mechanical separation between the tendon body and surrounding peritendinous tissues. Both Guardix‐SG and PM75 achieved significant downregulation of adhesion severity compared with PBS (*p* = 0.025 and *p* = 0.0029, respectively), with no significant difference between Guardix‐SG and PM75. Histological evaluation provided deeper structural differentiation between the two (Figure 7D). H&E and Masson's trichrome staining showed dense collagen deposition at adhesion sites in PBS‐ and Guardix‐SG‐treated rats, whereas collagen deposition was markedly attenuated in the PM75 group. Notably, the distinct histological appearance observed in the peritendinous space of PM75 group reflects localized cellular infiltration associated with active tissue remodeling and prolonged residence of the protective phospholipid matrix by anti‐inflammatory activity, rather than a classical pro‐inflammatory response. However, relative to normal tendon, both treatment groups manifested incomplete tissue restoration with hypertrophic changes at the primary injury site. Together, these results indicate that PM75 provides anti‐adhesion barrier performance comparable to Guardix‐SG in a tendon injury setting, whereas complete restoration of native tendon architecture may remain challenging in this model.

**FIGURE 7:**
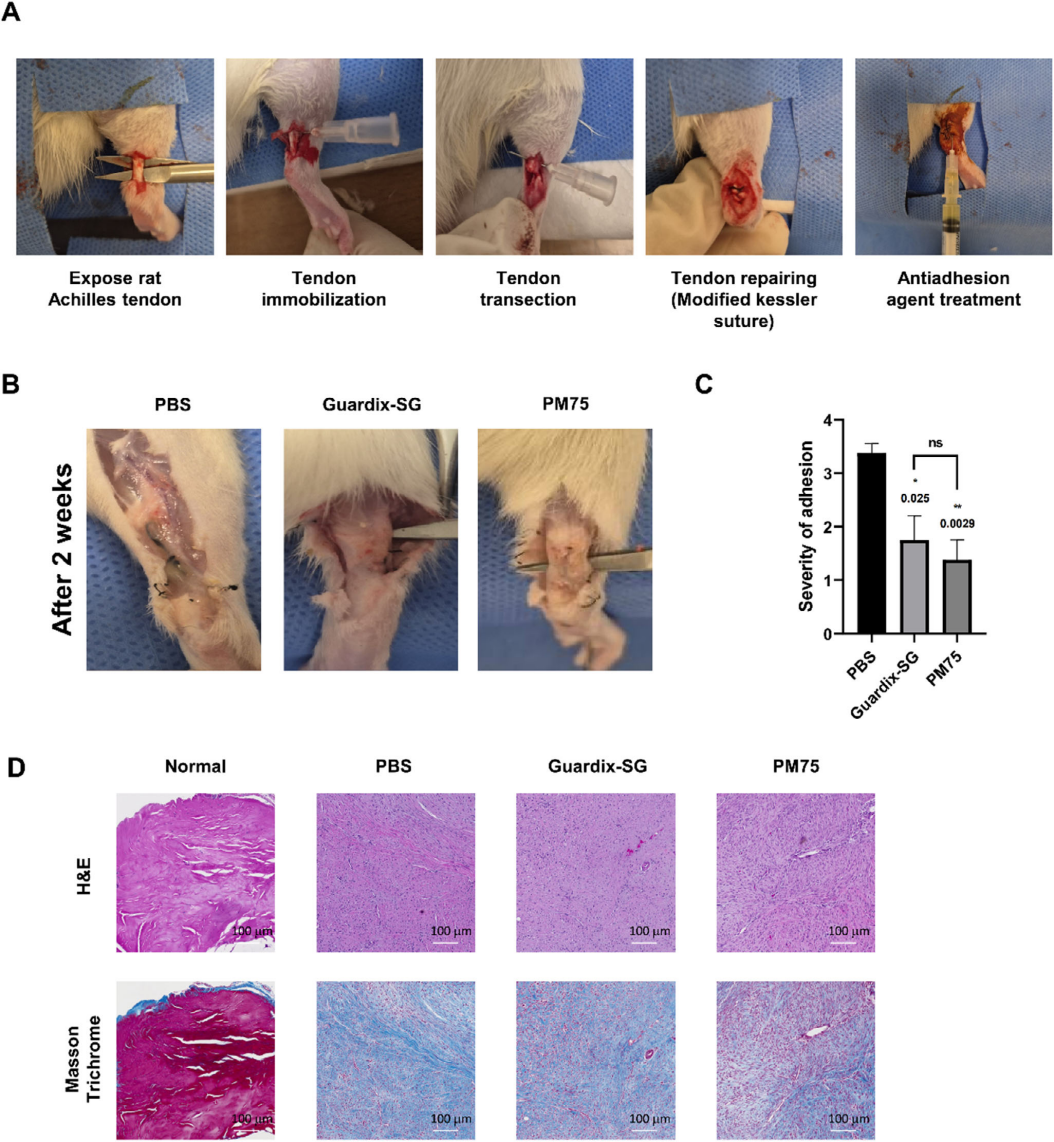
The therapeutic efficacy of PPSGs for tendon injury in rats. (A) The procedure of Achilles tendon injury model. (B) Representative gross images showing tendon adhesion prevention by PPSGs at Day 14 post‐surgery. (C) Quantification of tendon adhesion scores for severity at Day 14 post‐operation (*n* = 8). (D) Representative H&E and Masson's trichrome staining images of tendon adhesion sites on Day 14 post‐surgery. Data are shown as mean ± SE (C). Statistical analysis: Kruskal–Wallis test followed by Dunn's multiple‐comparisons post hoc test (C). *p < 0.05 and **p < 0.01.

## 4 Discussion

Postoperative adhesions remain a frequent and costly complication of surgical intervention. While existing commercial clinical barriers offer valuable therapeutic utility, their widespread clinical performance remains constrained by limited persistence and inconsistent structural retention at active injury sites. In this study, we converted PPSG, a depot‐forming phospholipid delivery system, into a drug‐free local barrier platform for postoperative adhesion prevention. This structural transition was fundamentally governed by solvent identity, which served as the primary design determinant regulating hydration‐triggered matrix assembly, underlying barrier mechanics, intraperitoneal residence, and overall tissue compatibility.

Substituting ethanol with propylene glycol markedly optimized water‐activated gel forming kinetics and macroscopic robustness of the barrier (Figure 2A–D). When compared with ethanol‐based formulations, propylene glycol‐based formulations demonstrated heightened and stronger hydration sensitivity and superior viscoelastic profiles. Prominently, PM75 exhibited gel‐like attributes even prior to hydration and further mechanical reinforcement upon water activation. SAXS analysis provided robust structural validation for this mechanical superiority, revealing highly organized long‐range lamellar packing in PM75 before and after hydration (Figure 2E,F). The higher‐order scattering peaks observed for PM75 suggest that propylene glycol supports a pre‐organized lamellar structure that is further reinforced through solvent–water exchange upon hydration. This denser lamellar arrangement directly underpins the reinforced gel network captured in rheological measurements. Importantly, this structural and mechanical reinforcement translated into enhanced functional utility at the tissue interface. PM75 showed higher adhesive strength on fresh rat abdominal wall tissue than both EM75 and Guardix‐SG. Although the dense internal matrix of PM75 required a higher injection force than the comparator formulations, the maximum force remained low (6.41 ± 0.24 N), below the standard 20 N threshold routinely used to define clinically relevant injectability for injectable hydrogel systems [38, 39]. Collectively, these findings indicate that propylene glycol‐based PPSG improves the balance between water‐activated barrier reinforcement, tissue adhesion, and syringe‐based applicability. Furthermore, the in vitro degradation profile showed that this reinforced gel architecture maintains its structural matrix for a prolonged period under aqueous conditions, a trend that was subsequently reflected by its prolonged intraperitoneal residence in vivo. Indeed, DiR‐based IVIS imaging indicated substantially prolonged intraperitoneal residence for PM75, whereas EM75 showed comparatively accelerated breakdown after the initial retention period, as indicated by the AUC analysis (Figure 3A–C). In the rat peritoneal‐cecum abrasion model, this mechanical and kinetic persistence corresponded to functional efficacy, with PM75 preventing macroscopic adhesions and reducing collagen deposition and clinical adhesion scores relative to control group (Figure 3E–I). To demonstrate translational generalizability beyond the peritoneal cavity, we evaluated PM75 in highly demanding rat Achilles tendon injury model. PM75 reduced peritendinous adhesions and showed performance comparable to Guardix‐SG by gross scoring, while histology suggested reduced collagen deposition in PM75‐treated tissues (Figure 7A–C). Because the peritendinous space is narrow, only a limited dose (0.1 g) could be locally administered, which likely constrained barrier coverage resulting in residual adhesions and hypertrophic remodeling in both groups. Yet, even under these stringent conditions, the reduced collagen deposition with PM75 may reflect not only barrier function, but also its intrinsic anti‐inflammatory activity.

Beyond serving as a physical separator, PPSG demonstrated a distinct, intrinsic immunomodulatory activity mode of action. In LPS‐stimulated macrophages, PPSG exposure shifted polarization away from a pro‐inflammatory phenotype and suppressed inflammatory cytokines at both transcript and translation levels (Figure 4C–H). Tissue‐level transcriptomic analysis further implicated the targeted down‐regulation of primary inflammatory programs, most notably NF‐κB activation. In alignment with these genetic insights, in vitro mechanistic assays confirmed that the material directly blocks macrophage supported attenuation of NF‐κB activation in macrophages, with pharmacologic re‐activation blunting the PPSG‐associated phenotype (Figure 5A–C). These data suggest that PPSG is not a passive coating but a material capable of reshaping the inflammatory‐to‐fibrotic trajectory that underlies adhesion maturation.

Several considerations and limitations should be noted when interpreting these findings and planning translation. First, while efficacy was demonstrated in both abdominal and tendon models, broader validation across additional injury severities, bleeding/exudate conditions, and longer follow‐up timepoints will be important to define the durability of benefit and potential recurrence. Second, although our data support NF‐κB‐linked immunomodulation and macrophage phenotypic shifts, the precise lipid‐derived molecular cues in the gel state remain to be elucidated, as do potential effects on other adhesion‐relevant cell types. Third, because Guardix‐SG was tested at a limited dose (0.5 g) in the peritoneal‐cecum adhesion model, which may be below its effective use range, its efficacy may have been underestimated. Accordingly, the limited protection observed for Guardix‐SG in the peritoneal model should be interpreted with caution. Last, given the intended single intraoperative application of PPSG, our safety and efficacy assessments were experimented on short‐term tolerability and barrier function until postoperative Day 10. However, long‐term safety evaluations, such as biochemical monitoring and late‐stage adhesion recurrence studies, will be essential in future investigations to support broader translational application.

Safety is a primary constraint for any barrier intended for local use at large doses. Ethanol‐containing formulations showed dose‐dependent cytotoxicity in vitro and signatures consistent with ethanol‐associated adverse effects in vivo, including liver injury markers and hemolysis at higher ethanol content (Figure 6D). In contrast, PM75 showed a favorable safety profile, including minimal hemolysis and no detectable abnormalities in major serum chemistry markers under the tested conditions (Figure 6E,F). These findings indicate that propylene glycol substitution is not only a performance optimization but also a safety‐enabling change for gram‐scale anti‐adhesion applications. Given the intended single intraoperative application, our safety assessment focused on short‐term tolerability through postoperative Day 10; however, longer‐term safety evaluation, including extended biochemical monitoring and quantitative immunological profiling, will be valuable in future studies, particularly to support broader translational scenarios and longer residence durations.

Although the present study focused on a drug‐free anti‐adhesion strategy, the intrinsic properties of PPSG may also allow its extension toward local drug depot applications. In preliminary studies, dexamethasone‐loaded PPSG formulations (PM50D and PM75D) showed sustained release for more than 15 days, supporting the potential future expansion of PPSG toward combined barrier and local drug‐delivery applications (Figure S8).

In summary, we establish PPSG as a clinically practical, water‐activated phospholipid barrier that can be translated from drug delivery to postoperative adhesion prevention. By replacing ethanol with propylene glycol, we enhanced gel mechanics and intraperitoneal residence while mitigating ethanol‐associated toxicity at gram‐scale dosing. Combined with phospholipid‐mediated immunomodulation that suppresses inflammatory and fibrotic progression, this PPSG system provides a dual‐action strategy through durable physical separation and local immune regulation, with strong potential for translational development as a safe and effective anti‐adhesion therapy.

## Author Contributions

**J.J.L**. and **Y.S**.: conceptualization. **J.J.L**., **Y.S**., **Y.K**., **H.Y**., **S.P**., and **B.Y**.: methodology. **J.J.L**. and **Y.S**.: investigation. **S.J.H**.: supervision. **J.J.L**., **Y.S**., and **S.J.H**.: writing – original draft. **J.J.L**., **Y.S**., and **S.J.H**.: writing – review and editing.

## Funding

National Research Foundation of Korea (NRF) funded by the Korean government (MSIT). Mid‐Career Researcher Program (NRF‐2021R1A2C2008834). National Research Foundation of Korea (NRF) funded by the Korean government (MSIT). Basic Research Infrastructure Support Program (University‐Centered Labs: RS‐2018‐NR031048). National Research Foundation of Korea (NRF) funded by the Korean government (MSIT). Bio & Medical Technology Development Program (NRF2022M3A9G8016257).

## Conflicts of Interest

The authors declare no conflicts of interest.

## Supporting information




**Supporting File**: adhm71710‐sup‐0001‐SuppMat.docx.

## Data Availability

All data are available in the main text or the Supporting Information. All RNA‐seq data generated in this study have been deposited in the GSE database under accession code GSE319081.
