# Supplementary material for: Water‐Activated Phospholipid Phase‐Separation Gel as an Immunomodulatory Anti‐Adhesion Barrier for Postoperative Adhesion Prevention
Source: Adv Healthc Mater. 2026 Sep 9;15(38):e71710. doi: 10.1002/adhm.71710 (PMC13674173; doi:10.1002/adhm.71710)
Supplement: Supplementary file 1 [file ADHM-15-0-s001.docx]

Supporting Information

Title

Water-activated phospholipid phase-separation gel as an immunomodulatory anti-adhesion barrier for postoperative adhesion prevention

Authors

Jong-Ju Lee^1†^, Yulseung Sung^1,3†^, Yeji Kim^1^, Heesang Yang^1^, Sooyeon Pyo^1^, Byeol Yun^2^ and Sung-Joo Hwang^1*^

^1^Yonsei Institute of Pharmaceutical Sciences, College of Pharmacy, Yonsei University, Incheon, Republic of Korea

^2^Department of Integrative Biotechnology, Yonsei University, Incheon, Republic of Korea

^3^Present: Boston Children’s Hospital and Harvard Medical School, 300 Longwood Avenue, Boston, MA 02115, USA

^*^ Corresponding author. Email: sjh11@yonsei.ac.kr

^†^ These authors contributed equally to this work

**Preparation of the water-activated phospholipid phase-separation gel (PPSG)**

Egg phosphatidylcholine (Lipoid E 80) was dissolved in a 1:1 (w/w) mixture of medium-chain triglyceride (MCT) oil and propylene glycol (PG). Four loadings—10, 25, 50, and 75 wt%—were formulated and denoted PM10, PM25, PM50, and PM75, respectively. As a solvent control, EM50 was prepared by dissolving egg phosphatidylcholine to 50 wt% in a 1:1 (w/w) mixture of MCT oil and ethanol. Batches were made at 10 g scale. Crude mixtures were transferred to amber glass vials under N2 purging for 10 min and stirred at 60 °C and 250 rpm overnight. After cooling to room temperature, formulations were stored at 4 °C in amber vials until use.

**Water-activated gelation and viscosity measurements**

Macroscopic gelation and viscosity changes upon water activation were evaluated for PM10, PM25, PM50, PM75, and the solvent control EM50. Deionized water was added at 0, 1, 2, 5, 10, 15, 20, 30, 40, and 50 wt% relative to the formulation mass, followed by 10 s of spatula mixing to induce shear-assisted activation. Viscosity was measured at 200 rpm and room temperature using a rotational viscometer (DV2T-LV, Brookfield, USA). Spindles LV-61, LV-62, or LV-63 were selected as appropriate to maintain measurements within 10–90% torque.

**Oscillatory rheology of PPSG**

To compare gel properties with and without water activation, oscillatory measurements were performed on a stress–controlled rheometer (MCR 302e, Anton Paar, Austria) using a 25-mm parallel-plate geometry with a 1.0-mm gap at room temperature. Formulations included PM10, PM25, PM50, PM75 and the solvent control EM50. For “5% water” conditions, samples were activated as previous section. “0% water” controls followed identical handling without water addition. Given the weaker gelation of the ethanol-containing control, EM50 was additionally examined at higher water fractions (up to 30 wt%) to capture its gel response.

Amplitude sweeps were conducted from 0.1 to 100% strain at a constant frequency of 10 Hz to obtain the storage modulus (G′) and loss modulus (G″). The linear viscoelastic (LVE) window was defined a priori (0.1–0.5% strain), and the G′ plateau was summarized as the average G′ within the LVE.

**In vivo anti-adhesion efficacy study in a peritoneal-cecum adhesion model**

The anti-adhesion activity of PPSG was evaluated in a peritoneal–cecum adhesion rat model using adult male Sprague–Dawley rats aged 6 weeks (Raonbio, Republic of Korea), randomly assigned to six groups (n = 10 per group): PBS (negative control), EM50 (ethanol–MCT solvent control), and PPSG formulations (PM10, PM25, PM50, PM75). Under isoflurane anesthesia, the injury in the peritoneal membrane and cecum was induced as previously described 34. The PPSGs were applied 0.5g to fully cover the injured surfaces, after which the peritoneum and skin were closed with nylon sutures and the area was disinfected with povidone–iodine. On postoperative day 10, rats were euthanized and the abdomen re-opened; adhesions between the injured surfaces were assessed by Zühlke classification for severity and extent of adhesion scoring (Supplementary Table 1)35.

**Histological analysis**

The adhesion area tissue (injured parietal peritoneum–cecum) and major organs (liver, heart, lung, spleen, kidney) were harvested, fixed in 10% neutral buffered formalin (48 h), paraffin-embedded, and sectioned at 5 µm. Adhesion-site sections were stained with H&E and Masson’s. The major organs were stained H&E for safety assessment. Slides were imaged on slide scanner (OCUS40, Grundium) with identical acquisition settings; scale bars were embedded. Masson’s Trichrome images were quantified for collagen area fraction (%) using ImageJ.

**Serum profiles for biomarkers of liver and renal injury**

0, 4, 8, 24, 48, and 120 h after PPSG administration, blood was collected from each animal, and serum was obtained by centrifugation at 5,000 × g for 20 minutes. The concentrations of aspartate aminotransferase (AST), alanine aminotransferase (ALT), and blood urea nitrogen (BUN) were measured using commercial assay kits for AST (BioAssay Systems, EASTR-100), ALT (BioAssay Systems, EALT-100), and BUN (BioAssay Systems, DIUR-100) in accordance with the manufacturers’ instructions.

**Flow cytometry analysis of CD80 and CD206**

RAW264.7 cells were seeded at 1 x 10^6^ cells and incubated with DMEM media supplemented with 10% FBS. Cells were added with LPS (100ng/mL) and corresponding PPSG for 24 hours. Cells were detached with versine and collected through centrifugation at 900 x g for 5 minutes. Cells were stained with anti-mouse PE-Cy7 F4/80 (123113, Biolegend, 1:100), PE CD206 (141705, Biolegend, 1:100), and FITC CD80 (104705, Biolegend, 1:100) for 18 hours. Live and dead cells were excluded via viability stain (AB_2869405, BD Biosciences). Finally, the samples were collected and analyzed via flow cytometry (BD FACSAria III) and Flow Jo (BD Biosciences).

**In vitro release study**

To evaluate the release behavior of dexamethasone acetate (DXA), DXA-loaded PPSG formulations were prepared by incorporating 1.33 mg of DXA into 0.27 g of PM50 and PM75, denoted as PM50D and PM75D, respectively. The release study was performed using dialysis tubing (molecular weight cutoff, 10 kDa) immersed in 40 mL of phosphate-buffered saline (PBS, pH 7.4) at 37 °C with shaking at 70 rpm. At predetermined time points, 1 mL of release medium was withdrawn and replaced with an equal volume of fresh PBS. The concentrations of DXA in the release samples were analyzed using a Waters Alliance 2695 Separations Module (Waters Corp., MA, USA). The HPLC analysis was performed under the following conditions: a Waters C18 reversed-phase column (5 μm, 4.6 mm × 150 mm, 125 Å; Waters, Dublin, Ireland), with the mobile phase comprising acetonitrile and water in a 7:3 (v/v) ratio. The flow rate was set at 1.0 mL/min under isocratic conditions, and the detection wavelength was set at 254 nm. Prior to analysis, all samples were filtered through a 0.45 μm syringe filter. DXA concentrations were quantified using a calibration curve prepared under the same analytical conditions.

**Statistics & Reproducibility**

All the statistical analyses were performed with GraphPad Prism 7 software. The qRT‒PCR data were statistically analyzed via Student’s t test, and the graphs show the means ± SDs. For the animal studies, the experiments were replicated across multiple batches. The mice were randomized into groups and treated according to the experimental design. The animal study data were statistically analyzed via Student’s t test, and the graphs show the means ± SDs. No statistical methods were used to predetermine sample sizes, but the group sizes used were similar to those commonly applied in mouse studies. No data were excluded from the analyses. The detailed methods and p values used to assess statistical significance are described in the Figure legends and detailed descriptions of the methods.

Figure S1. Mechanical characterization of PPSG formulations. (A) Representative inverted-vial images of PPSG at 0%, 5%, and 30% added water, showing water-dependent gelation. (B) Apparent viscosity of PPSG at 0%, 5%, and 30% added water (n=3). (C) Viscosity profile of PPSG as a function of water content (1–50%) (n=3). (D-E) Rheological properties of PPSG from amplitude sweep tests at 10 Hz. (D), EM50 with 0, 5 and 30% added water (E), PM10-75 with 0 and 5 added waters.

Figure S2. Therapeutic efficacy evaluation of PPSG formulations. (A) Representative gross images showing adhesion prevention by PPSG at day 10. (B-C) Adhesion scoring for (B) severity and (C) area at day 10 post-operation in PBS (control), EM50, and PM10–PM75 groups.

Figure S3. H&E staining of adhesive tissues at the surgery site. (A) H&E staining at day 10 on the adhesive tissues of the surgery site of rats treated with PBS, EM50, EM75, Guardix-SG®, PM10, PM25, PM50, and PM75 as anti-adhesive agents.

Figure S4. Masson Trichrome staining of adhesive tissues at day 10. (A-E) H&E staining and Masson Trichrome staining at day 10 on the adhesive tissues of animals treated with PBS (A), EM75 (B), PM75 (C), EM50 (D), and Guardix-SG® (E) as anti-adhesive agents.

Figure S5. in vivo effect of PPSG on inflammation and fibrosis in day 2. (A-G) mRNA expression levels at Day 2 normalized to Day 0 levels of animal tissues on the region of surgery treated with PBS, EM75, or PM75 (n=5). Inflammatory cytokine levels of iNOS (A), TNF-α (B), IL-6 (C), and IL-1β (D) are shown. Fibrosis pathway related genes of PAI (E), TGF-β1 (F), TGF-β2 (G) are shown. All the data are presented as the means ± SDs. All p values were determined by Kruskal–Wallis test followed by Dunn’s multiple-comparisons post hoc test; * p<0.05 (A-G). Source data are provided as a Source Data file.

Figure S6. Histology of major organs upon application of PPSG. (A-E), H&E staining of liver (A), heart (B), lung (C), spleen (D), and kidney (E) of mouse treated with PBS, EM50, EM75, Guardix-SG®, PM10, PM25, PM50, and PM75 at day 10

Figure S7. Toxicity of EM50 formulation. (A) Hemolysis ratio of EM50, EM75, PM75, DW (positive control), and Saline (negative control) (n=3). (B, C) Blood levels of AST, ALT, and BUN were measured to assess the acute (n=10) (B) and chronic (n=5) (C) toxicity of EM50. All the data are presented as the means ± SDs. All p values were determined by Student’s unpaired two-tailed t test (B) and Kruskal–Wallis test followed by Dunn’s multiple-comparisons post hoc test (C); ** p<0.01 (B, C).

Figure S8. In vitro release study of dexamethasone loaded PPSG formulations. In vitro release profiles of dexamethasone acetate loaded PM50 and PM75 (PM50D and PM75D, respectively).

Table S1. Scoring criteria of adhesion severity

|  | **Peritoneal-cecum adhesion model** | | **Tendon injury model** |
| --- | --- | --- | --- |
| **Grade** | **Severity of adhesion** | **Extent of adhesion** | **Severity of adhesion** |
| **0** | **No adhesion** | **No adhesion** | **No adhesion** |
| **1** | **Tiny, filmly adhesions** | **<25% of initial injured area** | **Small amount of membrane adhesion** |
| **2** | **Dense adhesions that require tension to dissect** | **25-50% of initial injured area** | **Small loose adhesive band that is easy to separate** |
| **3** | **Dense adhesions that require resection using scissors** | **50-75% of initial injured area** | **Moderate adhesion, the tendon has a certain degree of activity** |
| **4** | **Other intraabdominal organs form extensive adhesions** | **75-100% of initial injured area** | **Severe extensive adhesion, poor mobility and no boundary between the tendon and the peritendinous tissue** |

Table S2. List of cell lines used

| **Cell Line** | **Source** | **Cat#** |
| --- | --- | --- |
| HPDE (human, female) | Dr. Ming-Sound Tsao | HPDE |
| MEF (mouse, embryo) | ATCC | SCRC-1008 |
| RAW264.7 (mouse, male) | ATCC | TIB-71 |

Table S3. List of antibodies used

| **Antibody Name** | **Ratio Used** | **Company** | **Cat#** |
| --- | --- | --- | --- |
| Mouse monoclonal anti-CD206 | IF: 1:200, WB: 1:1000 | Santacruz | sc-58986 |
| Rabbit polyclonal anti-CD80 | IF: 1:200, WB: 1:1000 | Santacruz | sc-9091 |
| Alexa488-conjugated 2nd goat anti-mouse | IF: 1:500 | Invitrogen | A-11001 |
| Alexa594-conjugated 2nd goat anti-rabbit | IF: 1:500 | Invitrogen | A-11012 |
| Rabbit monoclonal anti-phospho-NF-kappaB p65 (Ser468) | WB: 1:1000 | Cell Signaling Technology | #3039 |
| Rabbit monoclonal anti-NF-kappaB p65 | WB: 1:1000 | Cell Signaling Technology | #8242 |
| Rabbit polyclonal anti-IkappaB alpha | WB: 1:1000 | Cell Signaling Technology | #9242 |
| Rabbit monoclonal anti-phospho-IkappaB alpha | WB: 1:1000 | Cell Signaling Technology | #2859 |
| Mouse monoclonal anti-beta actin | WB: 1:1000 | Santacruz | Sc-69879 |
| PE-Cy7 anti-mouse F4/80 | FACS: 1:100 | Biolgened | 123113 |
| FITC anti-mouse CD80 | FACS: 1:100 | Biolegend | 104705 |
| PE anti-mouse CD206 | FACS: 1:100 | Biolegened | 141705 |
| Alexa488-conjugated 2nd goat anti-mouse | IF: 1:500 | Invitrogen | A-11001 |
| Alexa594-conjugated 2nd goat anti-rabbit mouse | IF: 1:500 | Invitrogen | A-11012 |

Table S4. qRT-PCR Primer List

| **qRT-PCR Primer** | | | |
| --- | --- | --- | --- |
| **Species** | **Gene** | **Primer** | **5’ to 3'** |
| Mouse | TNF-α | F | GGTGCCTATGTCTCAGCCTCTT |
|  |  | R | GCCATAGAACTGATGAGAGGGAG |
| Mouse | IL-6 | F | TACCACTTCACAAGTCGGAGGC |
|  |  | R | CTGCAAGTGCATCATCGTTGTTC |
| Mouse | IL-1β | F | TGGACCTTCCAGGATGAGGACA |
|  |  | R | GTTCATCTCGGAGCCTGTAGTG |
| Rat | iNOS | F | TGGAGCGAGTTGTGGATTG |
|  |  | R | CCTCTTGTCTTTGACCCAGTAG |
| Rat | TNF-α | F | ACCTTATCTACTCCCAGGTTCT |
|  |  | R | GGCTGACTTTCTCCTGGTATG |
| Rat | IL-6 | F | GAAGTTAGAGTCACAGAAGGAGTG |
|  |  | R | GTTTGCCGAGTAGACCTCATAG |
| Rat | IL-1β | F | TCCCTGAACTCAACTGTGAAATA |
|  |  | R | GGCTTGGAAGCAATCCTTAATC |
| Rat | PAI | F | CTGGTGAACGCCCTCTATTT |
|  |  | R | ATGGTGCTACCATCAGACTTG |
| Rat | TGF- β1 | F | CCGCAACAACGCAATCTATG |
|  |  | R | CTTCCCGAATGTCTGACGTATT |
| Rat | TGF- β2 | F | GAGTGGCTTCACCACAAAGA |
|  |  | R | CTCCAGCTCTTGGCTCTTATTT |
